# Self-Expandable Metal Stents for Persisting Esophageal Variceal Bleeding after Band Ligation or Injection-Therapy: A Retrospective Study

**DOI:** 10.1371/journal.pone.0126525

**Published:** 2015-06-22

**Authors:** Martin Müller, Thomas Seufferlein, Lukas Perkhofer, Martin Wagner, Alexander Kleger

**Affiliations:** Department of Internal Medicine I, Ulm University, Ulm, Germany; RWTH Aachen, GERMANY

## Abstract

**Background and Study Aims:**

Despite a pronounced reduction of lethality rates due to upper gastrointestinal bleeding, esophageal variceal bleeding remains a challenge for the endoscopist and still accounts for a mortality rate of up to 40% within the first 6 weeks. A relevant proportion of patients with esophageal variceal bleeding remains refractory to standard therapy, thus making a call for additional tools to achieve hemostasis. Self-expandable metal stents (SEMS) incorporate such a tool.

**Methods:**

We evaluated a total number of 582 patients admitted to our endoscopy unit with the diagnosis “gastrointestinal bleeding” according to our documentation software between 2011 and 2014. 82 patients suffered from esophageal variceal bleeding, out of which 11 cases were refractory to standard therapy leading to SEMS application. Patients with esophageal malignancy, fistula, or stricture and a non-esophageal variceal bleeding source were excluded from the analysis. A retrospective analysis reporting a series of clinically relevant parameters in combination with bleeding control rates and adverse events was performed.

**Results:**

The initial bleeding control rate after SEMS application was 100%. Despite this success, we observed a 27% mortality rate within the first 42 days. All of these patients died due to non-directly hemorrhage-associated reasons. The majority of patients exhibited an extensive demand of medical care with prolonged hospital stay. Common complications were hepatic decompensation, pulmonary infection and decline of renal function. Interestingly, we found in 7 out of 11 patients (63.6%) stent dislocation at time of control endoscopy 24 h after hemostasis or at time of stent removal. The presence of hiatal hernia did not affect obviously stent dislocation rates. Refractory patients had significantly longer hospitalization times compared to non-refractory patients.

**Conclusions:**

Self-expandable metal stents for esophageal variceal bleeding seem to be safe and efficient after failed standard therapy. Stent migration appeared to be a common incident that did not lead to reactivation of bleeding in any of our patients. SEMS should be considered a reasonable treatment option for refractory esophageal variceal bleeding after treatment failure of ligature and sclerotherapy and non-availability of or contraindication for other measures (e.g. TIPS).

## Introduction

Gastroesophageal variceal bleeding remains associated with a high short and long term mortality. Current therapeutic strategies have led to a mortality decrease from about 40% to 15% [1–3]. Improvements in patient-centered care have been implemented by the application of a multimodal treatment: Initial hemodynamic stabilization, accurately timed airway management for prevention of aspiration-associated complications, antibiotic prophylaxis and pharmacological vasoconstriction and cautious volume- and transfusion strategies are performed prior to an immediate endoscopic therapy. This led to a redefinition of the time of endoscopic intervention and thus to a door-to-scope time up to 15 hours [4, 5]. To date, different interventional strategies like band ligation and sclerotherapy are used. Nonetheless, in about 10% of patients these procedures fail, which is in terms of outcome equivalent to persistent bleeding [6]. Once patients are refractory to conventional endoscopic therapy, the individual prognosis is deteriorating dramatically, mostly because of additional complications of the underlying liver disease. Transjugular intrahepatic portosystemic shunt (TIPS) and rarely immediate orthotopic liver transplantation can be performed in selected patients for effective termination of bleeding or reduction of rebleeding risk [7]. However, TIPS–and even more liver transplantation–are not generally available in many institutions. Hence, therapy-refractory variceal bleeding often requires the implementation of rescue procedures such as mechanical compression (e.g. Sengstaken-Blakemore tube) as a bridging therapy [7, 8]. Given the rather limited retention period of such mechanical compression strategies, new rescue strategies are warranted.

Until now, self-expandable metal stents (SEMS) were mainly used for treatment of esophageal obstruction/strictures, leaks, perforation or even tracheoesophageal fistulas. The first cases of SEMS application for esophageal variceal bleeding were reported eight years ago. Since 2006, SEMSs constructed for variceal bleeding have become commercially available outside of clinical studies. An array of case studies has been reported with a particular focus on primary bleeding control rates [9–13]. However, in most studies detailed clinical background of the patients remains elusive. In addition, there are no studies on stent application for non-variceal bleeding in the esophagus [9–13]. Here, we report 11 cases of stent application in a tertiary medical center for esophageal variceal bleeding including detailed clinical background information on the treated cases.

## Methods

### Study design

582 patients were admitted to our endoscopy unit between May 2011 and March 2014 with a primary diagnosis of “gastrointestinal bleeding”, according to our diagnosis database. We have analyzed all patients with variceal bleeding that have received either standard therapy or consecutive stent placement as a rescue therapy for refractory esophageal variceal bleeding. Patients were retrospectively analyzed including their clinical history. Patients with other reasons for esophageal bleeding (e.g. Mallory Weiss lesions or esophageal ulceration) were excluded from the analysis. All protocols are consistent to the Helsinki declaration (as amended on 2013) and approval was obtained from the local ethics committee (approval No. 283/14). Patient’s data were immediately anonymized. All patients were refractory to standard therapy (pharmacologic reduction of portal pressure, or endoscopic band ligation/sclerotherapy). Collected clinical data are presented in **Tables 1** and **2**.

**Table 1:** Description of patient cohort with stent treatment.

	total	Mean (y/d)	Min (y/d)	Max (y/d)	SD (y/d)
Age (years)		64,2	43	79	12,4
Sex	8m 3f				
Duration of hospital stay (days)		17	6	42	13,2
Duration of Stent in situ (days)		12,1	5	24	4,4
	total	**%**			
Chirrosis	10	90			
Ethiology: EtOH	9	81			
Hepatitis B/C	1	9			
Kryptogenic	1	9			
Jak-Mutation (with portal vein thrombosis)	1	9			
Previos bleeding episode	5	45			
HCC	3	27			
Portal vein thrombosis	4	36			
OV	8	72			
GOV I/II	3	27			
Paquet Grade					
I	1	9			
II	2	18			
III	6	54			
IV	2	18			
Hiatal hernia (small)	4	36			

Description of patients with SEMS (Self Expandible Metal Stent) treatment: Tabular description of patients characteristics, particularly average age, sex, duration of Stent in situ and diseases etiology; EtOH = Ethanol. HCC = hepatocellular carcinoma (possible multiple mentions in disease etiology), OV = isolated esophageal distribution of varicose. GOV I/II = combined esophageal and gastric distribution of varicose. Small hiatal hernia: Hernia with < 3cm longitudinal diameter.

**Table 2:** Complementary therapy and complications.

	total	%
Terlipressin	7	63
Octreotide	4	36
Propranolol	7	63
PCC	4	36
Intubation	5	36
CPR	1	9
ICU	8	72
Catecholamines	3	27
Hemodialysis/-filtration	1	9
Pulmonary infection	3	27
Acute renal failure	3	27
Patients with blood transfusion	8	72
TIPS	2	18
Liver transplantation	1	9
**Stent dislocation**		
early proximal	2	18
early distal	2	18
late proximal	2	18
late distal	1	18
no dislocation	4	36
Stent induced Ulcus	2	18
Tracheal Obstruction	none	0
Unsucessful stent deployment	none	0
Bleeding at time of Stent in situ	1	9
Death within 42 days	3	27

Complementary therapy, bleeding- and SEMS-specific associated complications. Illustration of additional pharmacological and non-pharmacological therapy of study patients. PCC = prothrombin complex concentrate. CPR = Cardiopulmonary Resuscitation. ICU = Intensive Care Unit. TIPS = transjugular intrahepatic portosystemic shunt. SEMS = Self-Expanding Metal Stent. Early Dislocation: Dislocation observed within 24h. Late dislocation: Dislocation observed after 24h or at time of stent removal.

#### Selection criteria for SEMS implantation

Patients with the following criteria did not receive a SEMS: Suspected or diagnosed malignancy or stricture affecting the esophagus. History of radiotherapy of the chest or the esophagus. Any malignancy of the throat, larynx, trachea, bronchi or stomach (the stent and its application system are not constructed for esophageal dilatation). Esophago-respiratory fistula. Non-esophageal lesions as cause of current bleeding episode, especially isolated gastric varices. Patient´s weight of lower than 45 kg. The following patients were excluded from the SEMS analysis cohort: (i) Patients with other types of esophageal bleeding (ulceration due to reflux, Mallory Weiss lesions and others). (ii) Several cases where the initial suspicion “variceal bleeding” could not be confirmed in follow up endoscopies. (iii) Patients with isolated gastric varices.

#### Statistical analysis

Standard error of the mean (SEM) are indicated by error bars. For comparisons of two groups, levels of significance were calculated with the unpaired Student’s t test as stated in the figure legends. Significances were calculated with Prism5 (GraphPad, USA).

### Treatment procedure

#### Pre-endoscopic treatment

None of the patients were transferred to the endoscopy unit without previous hemodynamic stabilization: All Patients received–if not already executed by paramedics–large lumen i.v. access and cautious volume therapy with crystalloids (target mean arterial pressure >65mmHg). Coagulation disorders were treated with PCC (prothrombine complex concentrate) or fresh frozen plasma, depending on the state of volume deficiency. Any patient with suspected variceal bleeding received empirically either octreotide (until mid 2012) or terlipressin (after mid 2012), which was continued for 48 hours after the intervention. Antimicrobial therapy was started with ampicillin + sulbactame. Hemodynamically instable patients with insufficient response to volume-therapy, catecholamine dependence, reduced vigilance or repetitive blood regurgitation were transferred to a intensive care unit (ICU) where protective intubation was performed before endoscopy. All other patients were treated in a certified intermediate care unit after the first bleeding episode for at least 24 hours.

#### Endoscopy and stent positioning

Endoscopy was performed in all patients within 2 hours after hospital admittance. Patients monitoring was executed according to the German S3 guidelines “sedation in gastrointestinal endoscopy” [14] (examination with at least two medical doctors and one nurse). In primarily hemodynamic instable patients, endoscopy was performed at the ICU. All patients received a complete gastroduodenoscopy; thus, proximal and distal non-esophageal bleeding sources could be excluded. Preferentially, band ligation therapy was attempted; if appropriate–sclerotherapy was evaluated. SEMS-implantation was performed if pharmacotherapy and all endoscopic approaches failed or were not reasonable due to the anatomic situation or lacking chance of exact localization of the bleeding spot.

To ensure appropriate handling of the SEMS device for variceal bleeding all endoscopists at our unit are frequently trained on an artificial stent deployment model. Every endoscopist has repetitively trained the stent deployment (> 3 times) at this self-generated model under supervision of an expert before he is allowed to join our on-call service for endoscopic emergency treatment. Moreover, every endoscopist had to train stent application at least once a year. Patients received a fully covered SEMS (SX-Ela stent Danis; Ella-CS, Hradec Kralove, Czech Republic). Mid-Diameter: 25mm, End-Diameter 30mm, Length: 135mm. The stent system features atraumatic stent ends and radiopaque markers at both stent ends and the midpoint. The delivery system is 28 Fr in diameter and 60 cm in length. All stents in our study were placed with endoscopic assistance. Stent application was performed according to the manufacturer’s recommendation.

#### Procedures after stent positioning

The correct stent position and cessation of bleeding were controlled immediately after stent positioning. A “second look” endoscopy was routinely performed in all patients within 24-48h after primary endoscopy. Chest X-Ray examination was done in all patients within 2 hours after primary endoscopic examination. After intervention patients were immediately evaluated for secondary therapy options such as TIPS or liver transplantation if appropriate. Afterwards secondary bleeding prophylaxis was initiated with ß-blockers/nitrates depending on systemic MAP (mean arterial pressure).

## Results

### Study patients

Among the 582 patients admitted to our endoscopy unit with the primary diagnosis of “gastrointestinal bleeding”, 82 patients suffered from esophageal variceal bleeding. In 71 patients bleeding could be either controlled using standard therapy (n = 70) or contraindication for SEMS implantation was apparent (n = 1, malignant stenosis caused by laryngeal carcinoma).

**Fig 1:**
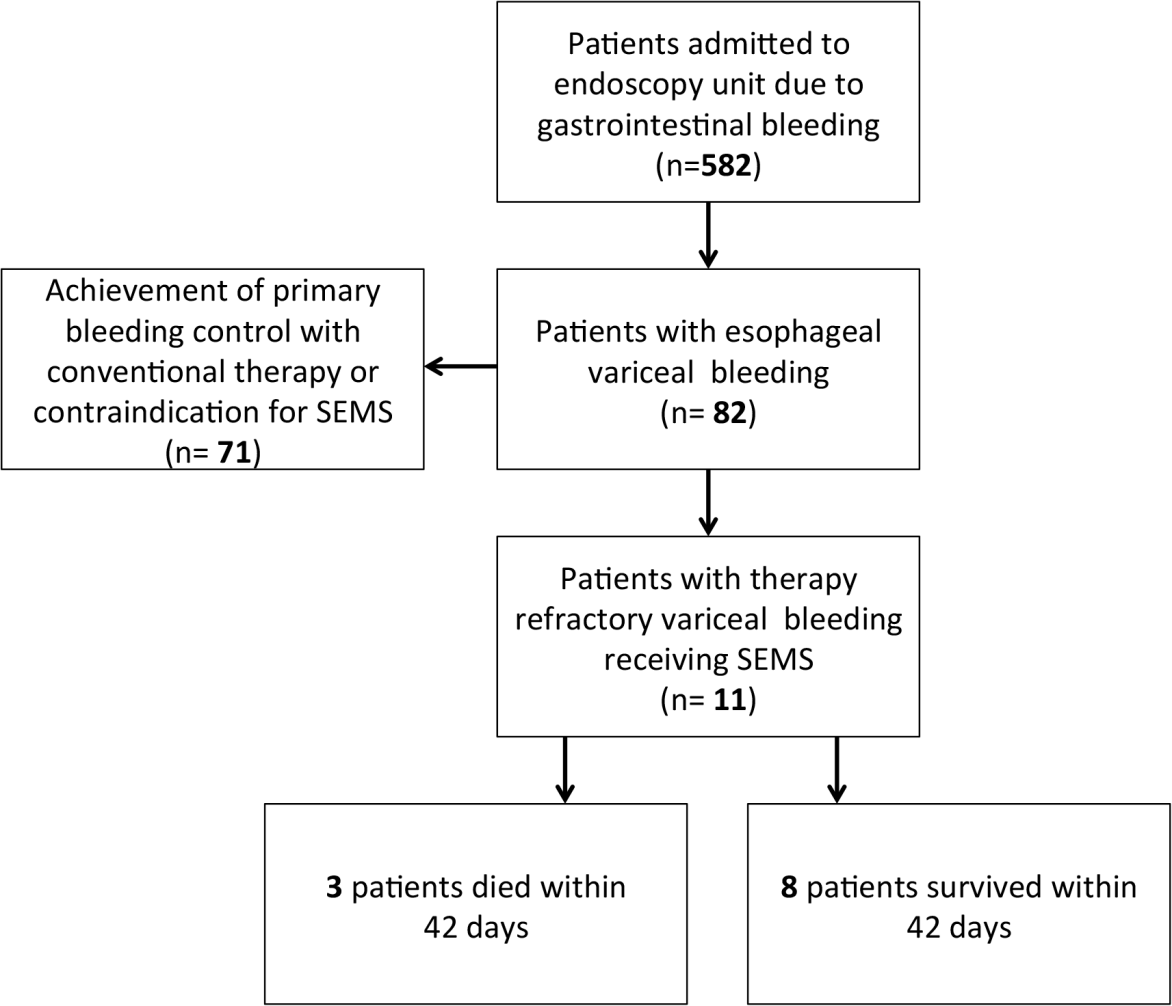
Enrollment and outcomes of study patients.

**Fig 2:**
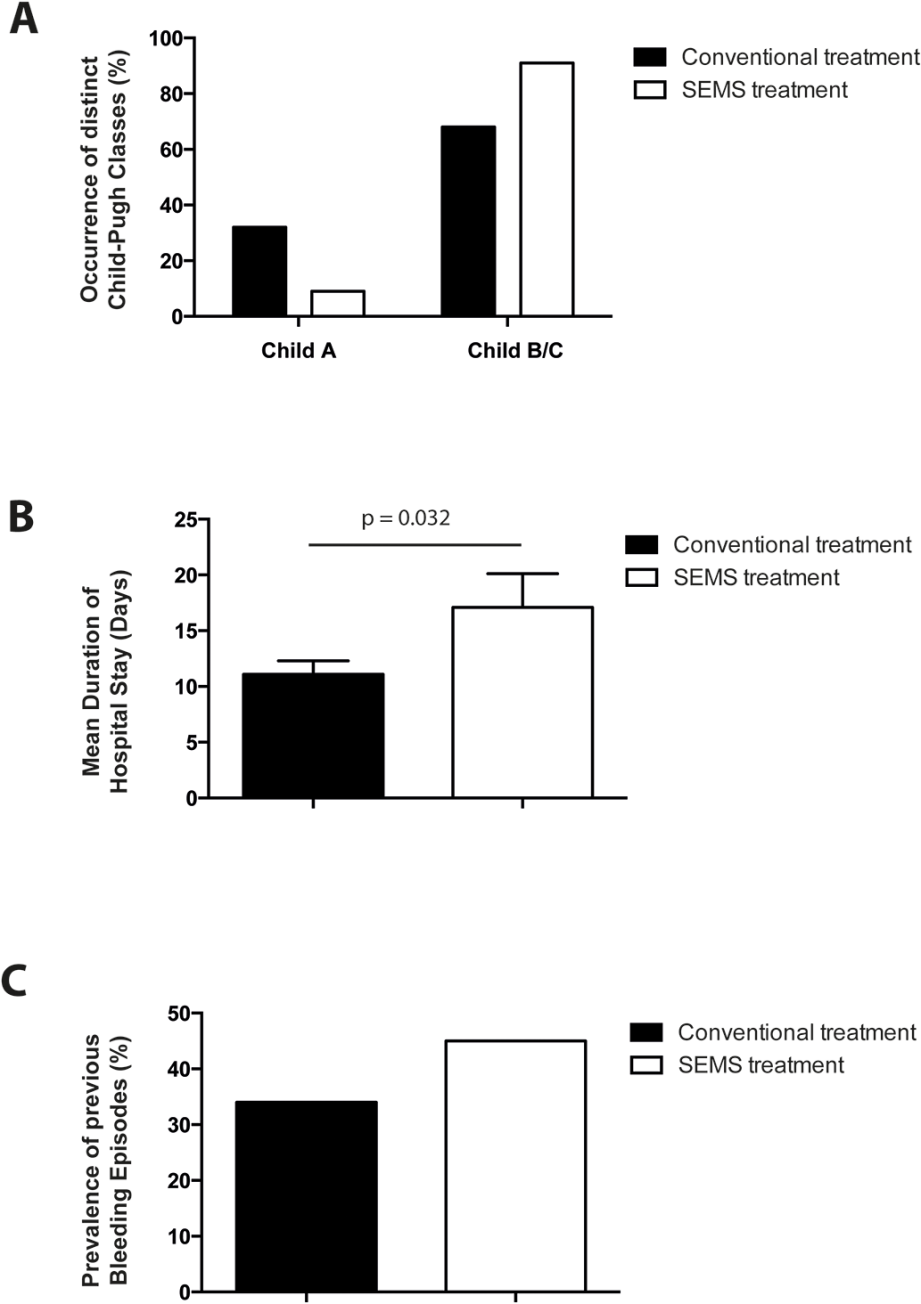
Differences in patients with variceal bleeding treated either with SEMS (Self Expandable Metal Stent) or without SEMS. (**A**): Occurrence of different Child-Pugh classes within the SEMS–group and the conventionally treated group. (**B**): Mean duration of hospital stay within the SEMS-group and the conventionally treated group, p-value was generated with an unpaired t-test. (**C**): Prevalence of previous bleeding episodes (%) in both groups.

11 cases (3 female, 8 male) were refractory to standard therapy leading to SEMS application (**Fig 1**). Average age of the patients was 64 years (range 43–72 years). 10 out of 11 patients suffered from advanced liver cirrhosis (Child-Pugh score B or C) from different origins (**Table 1, Table 3, Fig 2A**), (multiple reasons possible: alcoholic liver disease n = 9; hepatitis B n = 1, cryptogenic cirrhosis n = 1, portal vein thrombosis associated with a Jak2 mutation n = 1). Interestingly, patients with bleeding control upon conventional therapy had less advanced liver cirrhosis and fewer previous bleeding episodes (**Fig 2A** and **C**). Variceal localization was esophageal (OV) in 8 patients and gastroesophageal (GOV Type I/II) in 3 patients. Mostly, we found grade III varices (**Table 1**; Paquet classification: Grade I n = 1, Grade III n = 6, Grade II n = 2, Grade VI n = 2). In 4 individuals, portal vein thrombosis was an additional cause for the development of portal hypertension. Three patients in our study were suffering from hepatocellular carcinoma in addition to liver cirrhosis. We noticed ascites in the vast majority of the investigated patients (81.8%, n = 9). Compared to those patients receiving bleeding control with conventional treatment, amount of previous bleeding episodes was higher in the stent group (**Table 1**, **Table 4, Fig 2C**). In the SEMS cohort hemostasis was further compromised by coagulation disorder (n = 4) and/or low platelets (n = 8) (**Table 3**).

**Table 3:** Laboratory Findings.

**Patient No**	**INR D0**	**INR D3**	**AST D0 (U/l)**	**AST D3 (U/l)**	**Bilirubine D0 (µmol/l)**	**Bilirubine D3 (µmol/l)**	**Platelet Count D0 (Giga/l)**	**Patelet Count D3 (Giga/l)**
1	1,2	1,4	244	200	12	14	76	68
2	1,8	1,7	258	1457	23	61	108	51
3	1,3	1,3	130	183	43	89	88	121
4	1,3	1,3	163	101	18	18	131	71
5	1,3	1,3	21	24	23	23	121	63
6	1,2	1,6	111	67	31	26	101	91
7	3,9	1,7	219	2030	16	34	40	45
8	1,2	1,3	51	43	17	17	70	98
9	4,3	1,6	525	448	514	598	153	144
10	1,9	1,1	25	34	17	14	137	43
11	1,3	1,3	29	16	24	20	127	142
**Patient No**	**Creatinine D0 (µmol/l)**	**Creatintine D3 (µmol/l)**	**Hemoglobin D0 (g/dl)**	**Hemoglobin D3 (g/dl)**	**Hepatic Encephalopathy D3 (Degree)**	**Erythrocyte conzentrates (number of packed red cells)**	**Child Score**	**MELD-Score (at day of admission)**
1	62	60	8,8	7,6	0	4	non cirrhotic	non cirrhotic
2	168	150	8,2	8,2	II	8	C	20
3	130	134	9,3	8,9	I	2	B	17
4	59	53	12,4	8,7	II	2	B	10
5	119	130	11	8,6	0	4	A	13
6	127	111	9,8	9,7	I	4	B	14
7	150	338	7,7	6,5	III	4	C	27
8	53	69	10,7	11	II	0	B	8
9	78	97	13,4	14,6	III	0	C	36
10	67	52	10,2	7,4	III	6	B	14
11	192	118	8,5	7,9	II	0	B	18

Laboratory Findings, MELD- /Child Pugh-Score/ demand for blood tranfusion of study patients with SEMS (Self Expandible Metal Stent) treatment. D0 = Day of hospital admission. D3 = Day 3 after hospital admission. Chemical units in brackets.

**Table 4:** Patients with conventional treatment for variceal bleeding (n = 71).

	Mean (y/d)	Min (y/d)		Max (y/d)	SD (y/d)
**Age (years)**	62,5	34		86	13,5
**Duration of hospital stay (days)**	11	2		26	5,7
	**male**		**female**	
**Sex**	42		29	
	**total**		**%**	
**Number of patients with liver cirrhosis**	68		96	
**Cause of liver cirrhosis**				
EtOH	59		83	
Hepatitis B/C	6		8	
right ventricular failure/Cirrhose cardiaque	1		1	
others/kryptogenic	4		6	
**Number of patients with**				
Previos bleeding episode	24		34	
HCC/CCC	8		11	
Portal vein thrombosis	8		11	
OV	51		72	
GOV I/II	3		4	
JAK-Mutation	2		3	
**Paquet Grade**				
I	2		3	
II	13		18	
III	47		66	
IV	9		13	
**Child Score (cirrhosis in n = 68 patients)**				
**A**	22		32	
**B**	32		47	
**C**	14		21	

Description of patients with variceal bleeding and conventional treatment: tabular description of patients average age, sex, prevalence of cirrhosis, etiology of cirrhosis, prevalence of previous bleeding episodes, distribution of varices, Paquet Grade and Child-Pugh Score Classes.

### Bleeding control rate, stent positioning and stent dislocation

In none of our patients we observed persistent bleeding after stent deployment, leading to an immediate bleeding control rate of 100%. However, one patient required an additional intervention after 24 h due to a refractory variceal bleeding at the distal end of the stent. In this case, bleeding was controlled by a combined application of histoacryl injection and esophageal perivariceal sclerotherapy. Thus, the rebleeding rate within 48 hours was 9% (1 out of 11 patients). There was no rebleeding while the stent was *in situ* (5 to 24 days), neither in short term nor until stent extraction.

Stent dislocation after 1 day was a common event in our study group. Although correct stent positioning was verified within the first examination, we discovered stent dislocation in 7 out of 11 patients (63.6%). At the control examination after 24h, 4 patients showed stent dislocation (2 proximal, 2 distal). Interestingly, presence of a small hiatal hernia (<3cm longitudinal diameter) was not obviously linked to early stent dislocation events. Two of the patients with proximal dislocation had hiatal hernia, while the other two did not have herniation. Vice versa another 2 patients with correct stent position showed a small hiatal herniation. At the time of stent extraction another 3 patients had the stent placed outside of the correct location (2 proximal, 1 distal). However, there was no large hiatal hernia in the study cohort of 11 SEMS-receiving patients. Of note, complete dislocation to the stomach did not occur. Repositioning was technically easy, and in none of the patients we observed an induction of rebleeding by stent-repositioning (stent repositioning was performed in 4 patients, stent extraction in 3 patients of the dislocation group). In one patient rebleeding occurred during stent removal. Interestingly, in the 3 patients with gastric expansion of varices we did not observe treatment failure or bleeding reactivation of gastric varices, although SEMS seem to be more suitable for isolated esophageal varices (**Tables 1** and **2**).

### Additional treatment of study patients

Protective intubation was executed in 5 patients before endoscopy, while 8 out of 11 patients (72.7%) required endoscopy in an ICU setting. In one individual cardiac arrest occurred due to hypovolemic shock. After successful cardiopulmonary resuscitation, a SEMS was applied. Before stent application unsuccessful injection therapy with ethoxysclerol was attempted in 4 individuals. Histoacryl injection was used in one case of gastroesophageal varices. Given the requirement of a clear view for successful ligation therapy, none of the study patients received band ligation due to the massive hemorrhage. In the majority of cases (72.7%, n = 8), blood transfusion therapy was required. In the course of the hospital stay we applied on average 3.1 (range 0–8) units packed red cells per patient. Severely compromised coagulation was normalized by either fresh frozen plasma (in case of a volume depletion) or by substitution of prothrombine complex concentrates (PCC) in 4 out of 11 patients (**Table 2**).

### Bleeding associated complications and rebleeding rate within 42 days

Although primary hemostasis could be achieved in all patients, we observed prolonged duration of hospital stay in the study collective compared to those patients having with bleeding control under conventional therapies (mean duration 17 days vs. 11 days; **Tables 1** and **4**, **Fig 2B**). Worsening of hepatic encephalopathy was observed in most of the patients: 3 patients showed grade 3 and 4 patients grade 2 hepatic encephalopathy (HE), respectively. Initial bleeding associated hemoglobin (Hb) was an average of 10.0 g/l (range 7.7–13.4 g/l; SD 2.1), in virtually all patients we found a–partially marked–further decrease in hemoglobin, which made transfusion necessary in most patients. After 3 days averge hemoglobin levels were on average at 9.0 g/l (range 6.5–14.5 g/l, SD 2.2). In addition, 3 patients suffered from pulmonary infection or pneumonia. When reevaluating these patients for the presence of diagnostic criteria defining aspiration pneumonia, we noticed two out of three criteria: (i) New chest radiograph infiltrate and (ii) presence of risk factors for aspiration while (iii) infiltrates in a dependent pulmonary segment could not be detected on post-interventional chest x-ray films. Decrease of renal function with an increase of serum creatinine above 150 μmol/l or temporary anuria occurred in 3 patients, but was finally fully reversible. Most of the patients showed a temporary or persistent decompensation of their preexisting liver disease reflected by elevated liver enzymes and bilirubin levels (**Table 3**).

### Timing of stent removal

Appropriate timing of stent extraction has been discussed controversially in the literature with a wide range of stent placement duration [9–13, 15–18]. Early stent extraction might cause a reactivation of bleeding, while stent-associated ulcerations have been also reported [9–13]. However, in our study cohort 2 out of 11 patients (18.2%) showed stent-associated ulceration but there was no bleeding and therefore did not require further intervention. In our institution, stent removal was performed without the commercially available extraction kit. The average duration of stent disposition was 12.1 days, ranging from a minimum of 5 to a maximum of 24 days. Early stent removal was carried out in patients eligible for further treatment of portal hypertension, e.g. TIPS (transjugular intrahepatic portosystemic shunt), whereas in unstable patients with therapy refractory coagulation disorder stent duration was extended to our wide range of stent placement duration. In contrast to previously published retrospective analyses, none of our patients received band ligation directly after stent removal (**Table 2**).

### Mortality and clinical course

Despite successful primary bleeding control in all patients, variceal bleeding was still associated with a high risk of death within 42 days, 3 of 11 patients (27.3%) died within this follow up period. Only 1 patient died within the first week (day 5 after stent implantation, due to acute liver failure), none of the patients died because of uncontrolled bleeding. Two individuals received TIPS within 7 days after stent removal; one patient was transferred to a transplant center with subsequent successful liver transplantation. In the other patients we could identify contraindications for TIPS implantation (e.g. advanced hepatic encephalopathy) or TIPS procedure was refused.

## Discussion

To date, conventional treatment strategies contain a combination of pharmacological and endoscopic treatment. Still, between 5% and 15% of all patients with variceal bleeding cannot be sufficiently stabilized with the established treatment modalities [8]. Here, we describe our experience with a typical, critically ill patient collective that is not responding to standard therapy of variceal bleeding. We postulate that placement of SEMS appears to be a valuable option for refractory variceal bleeding providing advantages in terms of handling, timing and efficiency.

In line with previous studies, we observed an excellent primary bleeding control rate upon SEMS application and low rebleeding rates within the first 48 hours. Within this particular period, previous reports of conventional therapeutic approaches show the highest rates of variceal rebleeding [19]. Upon stent extraction, the bleeding rate within the next 24 hours was also low. In this context, SEMS appears superior to balloon tamponade. Avgerinos et al. reported 30 patients with non-refractory variceal bleeding primarily treated with balloon tamponade. They observed primary hemostasis, rebleeding rate after 24h and complications in 80%, 58% and 33%, respectively [20]. Similar numbers for the use of balloon tamponade were reported by other studies [21]. Tracheal compression due to stent implantation was also not observed, which goes along with the low incidence of this complication as described in other studies [9–13].

Although the manufacturer emphasizes the possibility of stent deployment without prior endoscopy, this opportunity was not described yet in studies or case reports. Previous reports indicate that this new tool can be “easily put in place” [9, 10]. To our experience, regular training is required to prevent procedural tentativeness, as the multi-step stent deployment under emergency conditions might be difficult when performed by an untrained investigator. Especially in this case, an illustrated point-by-point manual provided by the manufacturer cannot compensate frequent exercise. In fact, in most of the cases a guide wire was used to ensure proper stent positioning. Also the devices balloon with a diameter of approximately 6 cm (estimated at a volume of 100–120 ml) might not be able to counterforce for correct positioning in patients with bigger hiatal herniation. This is one of the reasons for us not to recommend a blind stent delivery as recommended by the manufacturer. Moreover blind delivery might lead to via falsa pulmonary damage and should therefore be limited to ultima ratio situations with absence of an endoscopic facility. Thus, regular training sessions at tertiary centers and endoscopic stent deployment control should be mandatory to guarantee appropriate handling and placement by inexperienced endoscopist for whom the device could be particularly helpful. In turn, from our point of view SEMS remains a device reserved for interventional endoscopists.

In our study cohort the maximum stent time was 24 days while in the literature also longer time intervals have been reported. These variations are most likely due to the heterogeneity of underlying liver disease [8–13, 15–18]. Thus, SEMS seems to be a suitable device in terms of bridging to definite therapy such as transplantation or TIPS.

Interestingly, we observed high rates of primary stent dislocation mostly after 24 hours suggesting inappropriate positioning of the stent at the first intervention. Therefore, we would recommend to perform early control endoscopy for the following reasons: (i) To ensure correct stent localization for persistent hemostasis, (ii) a correct stent position enables liquid oral nutrition, (iii) the repositioning of the stent in our hand never led to reactivation of bleeding and thus supports the afore mentioned reasons. (iv) Finally, we observed a relevant proportion of stent migration events at the time of stent extraction. Thus, early repositioning may help to reduce such migration events due to improved localization under controlled endoscopy conditions.

Most importantly, incidence of stent ulcera did not correlate with a prolonged stent *in situ* time (8 days and 16 days in the 2 mentioned patients). Given the high relapse rates of variceal bleeding within the first 5 days [8, 19], this explains the much higher rebleeding rates in case of the balloon tamponade. In case the maximum inflation time is not exceeded, balloon tamponade-associated complications are also minor and limited to pulmonary infections and nasal excoriation [21–23]. Also in our cohort we noticed a relevant proportion of pulmonary infection. This is most likely not linked due to the device but instead due to massive hemorrhage leading to a high likelihood of aspiration. Albeit not evaluated in our patients, it is likely that the discomfort for a patient of having a SEMS in situ is far lower than in case of balloon tamponade (discomfort rate of 100%, [21])

The high primary success rates using SEMS have to be balanced against a persistently high morbidity and mortality rate in our study cohort but also in other studies reporting 6-week mortalities between 30% and 77% [10, 15, 18, 24]. In line, only two patients could be dismissed within the first 10 days of hospital stay underpinning our observation of a prolonged hospital stay in refractory patients compared to patients receiving standard of care. In fact, our cohort comparison indicates as expected that more advanced liver cirrhosis stage and number of previous bleeding episodes increase risk for refractory variceal bleeding and thus SEMS requirement. Thus, causal therapy for portal hypertension is urgently needed for these patients such as liver transplantation or TIPS application. This points to the option of complementary use of SEMS in the initial bleeding event and TIPS as long term therapy. In line, in those patients that were not eligible for TIPS or liver transplantation, we observed a high risk of morbidity and death [8].

In summary, we provide with the current retrospective study a series of successful applications of SEMS in cases of refractory variceal bleeding of the esophagus. Thus, our study is in line with previous reports and confirms a high success rate of this procedure. To date, essential disadvantages of the system are the high cost for a single stent, the stent positioning system, which requires regular teaching sessions, and frequent stent dislocations.
